# Growing Up, Hooking Up, and Drinking: A Review of Uncommitted Sexual Behavior and Its Association With Alcohol Use and Related Consequences Among Adolescents and Young Adults in the United States

**DOI:** 10.3389/fpsyg.2019.01872

**Published:** 2019-08-22

**Authors:** Tracey A. Garcia, Dana M. Litt, Kelly Cue Davis, Jeanette Norris, Debra Kaysen, Melissa A. Lewis

**Affiliations:** ^1^Department of Psychology, Murray State University, Murray, KY, United States; ^2^Department of Health Behavior and Health Systems, School of Public Health, University of North Texas Health Science Center, Fort Worth, TX, United States; ^3^Edson College of Nursing and Health Innovation, Arizona State University, Phoenix, AZ, United States; ^4^Alcohol and Drug Abuse Institute, University of Washington, Seattle, WA, United States; ^5^Department of Psychiatry and Behavioral Sciences, University of Washington, Seattle, WA, United States

**Keywords:** hooking up, alcohol, adolescents, young adults, review

## Abstract

Hookups are uncommitted sexual encounters that range from kissing to intercourse and occur between individuals in whom there is no current dating relationship and no expressed or acknowledged expectations of a relationship following the hookup. Research over the last decade has begun to focus on hooking up among adolescents and young adults with significant research demonstrating how alcohol is often involved in hooking up. Given alcohol’s involvement with hooking up behavior, the array of health consequences associated with this relationship, as well as its increasing prevalence from adolescence to young adulthood, it is important to determine the predictors and consequences associated with alcohol-related hooking up. The current review extends prior reviews by adding more recent research, including both qualitative and experimental studies (i.e., expanding to review more diverse methods), research that focuses on the use of technology in alcohol-related hookups (i.e., emerging issues), further develops prevention and intervention potentials and directions, and also offers a broader discussion of hooking up outside of college student populations (i.e., expanding generalization). This article will review the operationalization and ambiguity of the phrase hooking up, the relationship between hooking up and alcohol use at both the global and event levels, predictors of alcohol-related hooking up, and both positive and negative consequences, including sexual victimization, associated with alcohol-related hookups. Throughout, commentary is provided on the methodological issues present in the field, as well as limitations of the existing research. Future directions for research that could significantly advance our understanding of hookups and alcohol use are provided.

## Introduction

In the United States, adolescence and young adulthood are times marked by self-exploration, instability, decision making, and identity formation (Arnett, 2000, 2004; Manning et al., 2006; Arnett et al., 2011). These explorations frequently occur within the context of romantic and sexual relationships (Osgood et al., 2005; Shanahan et al., 2005; Settersten, 2007) as well as alcohol use. Among United States adolescents in grades 9–12, 39.5% report having ever had sex and the mean age at first vaginal sexual intercourse among United States adolescents is 17.1 (Substance Abuse and Mental Health Services Administration (SAMHSA), 2011; Centers for Disease Control and Prevention [CDC], 2018). While most first sexual experiences occur within relationships, roughly 2/3 of adolescents who are sexually active have sexual experiences with partners they are not dating (Manning et al., 2000, 2005). Moreover, romantic and/or sexual relationships among adolescents and young adults can be transitory (Cohen et al., 2003; Foxman et al., 2006; Bogle, 2008; Lyons et al., 2014). In addition to sexual experiences often occurring in adolescence and young adulthood, alcohol use initiation and continuation occurs during these developmental periods and research indicates that sexual experiences and alcohol use often co-occur (Johnson and Chen, 2015).

The co-occurrence between alcohol use and sexual behavior is not surprising as alcohol use is prevalent in both adolescence and young adulthood with 9.4% of those aged 12–17 and 57.75% of those aged 18–25 engaging in past-month alcohol use (Substance Abuse and Mental Health Services Administration [SAMHSA], 2016). By 12th grade, approximately 71.7% of adolescents have consumed alcohol (Kann et al., 2018). Further, there appears to be an age-graded trend with alcohol use increasing from adolescence into young adulthood (Jackson and Sartor, 2016). This indicates that at the same time adolescents and young adults are exploring their sexual and romantic identities, they are also beginning to drink alcohol more frequently. Indeed, hooking up and alcohol use do not only seem to co-occur, but in many young adults’ and adolescents’ schemas regarding hooking up, alcohol is involved (e.g., Holman and Sillars, 2012; Livingston et al., 2012). Importantly, the use of alcohol in sexual encounters can increase the likelihood that individuals experience both emotional (i.e., depression, guilt, shame) and physical risk (i.e., sexually transmitted infections, unwanted pregnancies) (e.g., Weinstock et al., 2004; Lewis et al., 2012); thus, research with a focus on sexual behaviors and alcohol use throughout adolescence and young adulthood is of critical importance.

Although there are many possible frameworks (i.e., viewing hooking up as a risk behavior, as a biological imperative, as a new cultural norm, or as an aspect of personality) that could be used to guide research examining the association between hooking up and alcohol use and related consequences, alcohol myopia theory has been described as the most important theory for understanding the association between alcohol use and sexual risk-taking and sexual victimization (Kaly et al., 2002). According to alcohol myopia theory (Steele and Josephs, 1990), alcohol disinhibits behavior primarily as a result of its pharmacologic effects on information processing, such that acute alcohol intoxication creates cognitive impairment that results in an inability to attend fully to situational cues. For risky sexual behavior, acutely intoxicated individuals may be less likely to attend to less salient and more distal cues (i.e., STI risk) and more likely to attend to more salient and proximal cues (i.e., sexual arousal in the moment) resulting in a greater likelihood of risky sexual behavior, such as hooking up (Davis et al., 2007; Rehm et al., 2012; Scott-Sheldon et al., 2016). Regarding sexual victimization, acute alcohol intoxication can cause cognitive impairments that can lead to ineffective risk perception and lower likelihood of using effective resistance strategies (Norris et al., 2006; Stoner et al., 2007; Davis et al., 2009; Testa and Livingston, 2009). Both survey and experimental studies have demonstrated a connection between alcohol use and both risky sexual behavior and sexual assault (e.g., Parks et al., 2008; Krebs et al., 2009; McCauley et al., 2010; Patrick et al., 2012; Rehm et al., 2012; Testa and Hoffman, 2012; Howells and Orcutt, 2014; Scott-Sheldon et al., 2016). Given that alcohol myopia has been applied broadly to sexual risk-taking in general (e.g., Kaly et al., 2002), it is an appropriate framework with which to guide the current review. As such, the present review will focus on one specific set of sexual behaviors, hooking up, in terms of definitions, predictors, and its association with alcohol use and related consequences among adolescents and young adults, with the understanding that alcohol myopia is one potential mechanism.

In order to ground this review, we have chosen a definition of hooking up in line with other studies (e.g., Lewis et al., 2012, 2013; Fielder et al., 2013), such that we define a hookup as an uncommitted sexual encounter that may or may not include intercourse, which occurs between individuals in whom there is no current dating relationship and no expressed or acknowledged expectations of a relationship following the encounter. Estimating the prevalence of hooking up is more difficult among adolescents compared to young adults, given the limited work on hooking up specifically (as opposed to sexual risk behavior more generally); however, some estimates in the United States indicate that hooking up occurs among approximately 26–28% of adolescents (Fortunato et al., 2010; Lyons et al., 2014), and three-fifths of sexually active adolescents experience casual sex before young adulthood (Manning and Lyons, 2009). Among young adults in the United States, there is substantially more research on hooking up with rates ranging from 67 to 86% (Armstrong et al., 2009; Garcia et al., 2012; Fielder et al., 2013). Additionally, hooking up seems to follow an age-graded trend which increases from adolescence into young adulthood and peaks around the age of 21 (Lyons et al., 2015). This trend is not surprising given the developmental tasks of young adulthood whereby committed relationships can be a seen as a challenge to individual life plans in the realms of education, work, and financial demands (e.g., Shulman and Connolly, 2013; Spell, 2016).

Studying hooking up has expanded in the last several years across many research areas and alcohol use is one behavior commonly linked with hooking up. The alcohol use-hooking up relationship has been found at the global level (i.e., general correlations between alcohol use and hooking up) (e.g., Grello et al., 2006; Owen et al., 2010; Lewis et al., 2012; Fielder et al., 2013; Olmstead et al., 2013b), the event level (i.e., both behaviors occurring during specific events) (LaBrie et al., 2014), and in qualitative research (i.e., data collected through interviews or observation) (e.g., Downing-Matibag and Geisinger, 2009; Spell, 2016; Richman et al., 2017). While hooking up overall may not be necessarily risky in and of itself, it is of particular importance to study the effects of alcohol use with hooking up given their high prevalence rates and links to negative psychological consequences (including depression and low self-esteem, e.g., Fielder and Carey, 2010a), physical health consequences (i.e., STI and pregnancy risk), associations with other risky sexual behaviors (e.g., multiple partners, inconsistent condom usage), and sexual victimization.

Given that hookups can be unplanned, with unfamiliar partners, and in unfamiliar contexts, the occurrence of unprotected sex may increase. Associations related to unprotected sex are important to investigate as adolescents and young adults have the highest rates of gonorrhea, chlamydia, and undiagnosed HIV and account for half of the 19 million new cases of STIs diagnosed in the United States each year (Centers for Disease Control and Prevention [CDC], 2016). A greater understanding of the relationships among alcohol, hooking up, and related consequences will provide insight to how and for whom prevention programs should be targeted to reduce possible negative consequences. Additionally, because promoting safer sexual behavior is one of the U.S. Department of Health’s *Healthy People 2010* goals (United States Department of Health and Human Services and National Center for Health Statistics, 2001) and will continue to be a leading health indicator for *Healthy People 2020* (U.S. Department of Health and Human Services, 2011), developing a greater understanding of how to promote safer sexual practices under the circumstances of hooking up is imperative. As such, this article will review terminology used to describe hooking up and will describe the associations between hooking up and alcohol use, predictors of alcohol-related hooking up, positive and negative consequences of alcohol-related hooking up, and future directions for research.

In order to achieve the aims of this review, studies were retrieved from (1) electronic databases, (2) reference sections of relevant papers, and (3) professional journals using established guidelines (Rosenthal, 1991; Reed and Baxter, 2009). First, we searched electronic databases (PubMed, PsycInfo, ERIC, Dissertation Abstracts, The Cochrane Library) using a search strategy that included the terms “hooking up,” “hookup,” “risky sexual behavior,” “casual sex,” “friends with benefits,” “one-night stands,” “adolescents,” “young adults,” “adolescence,” “college students,” “emerging adulthood,” “uncommitted sex,” “casual sexual relationships and experiences,” and “alcohol” up until December 1, 2018. Search terms were adjusted based on the specific requirements of each electronic database. We conducted a broad search of studies examining alcohol-related hooking up. Second, we reviewed the references of papers obtained through the database searches. Third, we examined the tables of contents and/or abstracts available from relevant electronic journals (e.g., *Journal of Sex Research; Archives of Sexual Behavior; Addictive Behaviors, Journal of Studies on Alcohol and Drugs; Psychology of Addictive Behaviors*). Titles and abstracts for all references were reviewed with relevant articles retained for full paper reviews. The literature cited within this paper was chosen with a focus on understanding the current literature on hooking up and alcohol use among adolescents and young adults and not on risky sexual behavior generally. Articles were discarded if they did not focus explicitly on both alcohol use and hooking up. For example, although there is a broad literature on hooking up in general, only studies that focused on the overlap between alcohol and hooking up were retained for the review. Similarly, articles focusing on alcohol use and sexual behavior generally were discarded if they did not explicitly address hooking up. Initial searches revealed approximately 150 potential studies published before April 1, 2019. Of these studies, titles and abstracts were reviewed and 59 articles were retained for full paper review.

It is important to note that there have been previous reviews of hooking up (Heldman and Wade, 2010; Garcia et al., 2012), sexual risk behaviors, and alcohol use (Brown et al., 2016), and a meta-analysis of quantitative studies on hooking up and alcohol use (i.e., Claxton et al., 2015). While these reviews and meta-analysis propose several directions for future research and added to the existing body of research on these topics, our review specifically focuses on the relationship between hooking up and alcohol use, not sexual behavior more generally or hooking up only. Additionally, the meta-analysis conducted by Claxton et al. (2015) proposed several directions for future research on hooking up and alcohol use; specifically, the need for more comprehensive and consistent study of hooking up and alcohol use, for the use of diverse methods (including longitudinal studies), and for research outside of homogeneous college populations. While this meta-analysis was an important step in establishing a link between alcohol use and hooking up, the current review adds to the literature by incoporating more recent research, including both qualitative and experimental studies (i.e., expanding to review more diverse methods), research that focuses on the use of technology in alcohol-related hookups (i.e., emerging issues), further develops prevention and intervention potential and directions, and also offers a broader discussion of hooking up outside of college student populations (i.e., expanding generalization).

## Operationalizing Hooking Up

Before delving into a review of the alcohol and hooking up literature, it is important to first define hooking up. In reviewing the literature on hooking up, it is notable that there are a number of related terms, e.g., casual sex, friends with benefits, one-night stands, uncommitted sexual encounters. However, not all of these terms are used to describe the same type of sexual behavior or have the same risks or associations. While this review focuses on hooking up and its association with alcohol use and related consequences among adolescents and young adults, it is essential to clarify what hooking up is *not* in order to get a clear sense of what exactly the term refers to. For instance, hooking up is distinct from dating in that in dating typically involves some form of romantic relationship between two people whereas no current or future relationship is present or expected among two individuals hooking up (Glenn and Marquardt, 2001; Bogle, 2008). Also of importance, it is essential to note that many of these terms are not mutually exclusive. For example, a hookup and a one-night stand might be one and the same; however, they may also differ in that penetrative sex is typically expected in a one-night stand whereas hooking up can involve sexual contact ranging from kissing to penetrative sex.

The term hooking up appeared in the literature after 2000 (e.g., Paul et al., 2000; Glenn and Marquardt, 2001). Since this time, there have been numerous definitions for the term, which has the potential to lead to problems in research (i.e., difficulty comparing findings across studies, inaccurate rates of the behavior or associated risk). Definitions of hooking up found in the literature often vary by what is considered uncommitted and with whom hookups occur. For example, level of commitment may be specific for the current relationship at the time of the hookup (i.e., not currently dating, not currently friends or previous romantic partners) or commitment following the hookup. Regarding hookup partners, some definitions of hooking up have been limited to heterosexual experiences or only new partners who are strangers or brief acquaintances, thus omitting previous partners, friends, or same-sex sexual behaviors (Paul et al., 2000; Glenn and Marquardt, 2001). It is important to note that while most definitions now include both same- and opposite-sex experiences, research outside of White, heterosexual college student populations is still sorely needed (Rupp et al., 2014).

While researchers historically have not used a consistent definition of hooking up, the field is generally working toward standardization of terminology. A clearer conceptualization of hooking up is more the result of researchers wanting to increase meaning in study findings rather than a reflection of cultural changes in hooking up. Recent definitions of hooking up found in the literature tend to address: (1) the non-committed nature of the hookup, such that there are no expressed or acknowledged expectations of a relationship following the hookup, (2) that hookups include sexual contact ranging from kissing to penetrative sex, and (3) that hookups may occur between two people who are strangers, acquaintances, friends, or ex-partners (Garcia et al., 2012; Olmstead et al., 2013b). Moreover, the use of other terminology to describe the same boundaries of behavior occurs as well (e.g., Claxton and van Dulmen, 2013; Claxton et al., 2015; Boislard et al., 2016; refer to this set of behaviors as casual sexual relationships and experiences). However, it should be noted that using research-generated definitions for hooking up does not resolve all possible methodological issues. For instance, participants may disregard the provided definition in research and answer questions based on their personal understanding of the term, which may vary widely (Epstein et al., 2009; Fielder and Carey, 2010a; Claxton and van Dulmen, 2013; Lewis et al., 2013). The field would greatly benefit from consistent use of a standardized definition of hooking up to better draw comparisons across studies and ease interpretation of findings when examining how hooking up is associated with other health and risk outcomes.

## Hooking Up Ambiguity

In addition to (and perhaps as an analog of) less than optimal operationalization of the term hooking up among researchers, it is notable that young adults have various meanings for the term hooking up (e.g., Bogle, 2008; Epstein et al., 2009; Holman and Sillars, 2012; Currier, 2013; Lewis et al., 2013; Olmstead et al., 2017). Lewis et al. (2013) completed a study in which a random sample (*N* = 1,468) of undergraduate students (56% female) provided in their own words what the term hooking up meant to them. The research team coded and analyzed these open-ended definitions of hooking up and determined that there were three clusters of student definitions. Cluster 1 had the broadest definition of hooking up and included sex generally, making out, and confusion over the term hooking up. Cluster 2 had a definition that noted sex generally but that also placed emphasis on interpersonal and social aspects of hooking up (i.e., way to meet people, parties). Finally, Cluster 3 had a definition that focused on sex with notable reference to specific types of sex (i.e., oral, digital). It is clear from these findings that the ambiguity of the term leaves it open to interpretation among college students. There is some evidence that adolescents may vary in their description of hooking up by age. In one qualitative study, hooking up was associated with “having sex” among high schoolers, but not among middle schoolers. Middle school students likened hooking up more often with dating behavior; however, some high school students did as well (Rowley and Hertzog, 2016). While it would seem that researchers who examine hooking up have a goal to move toward a consensus about how best to define hooking up, adolescents and young adults may have no desire to do so based on the potential of positive consequences resulting from the phrase’s ambiguity (Currier, 2013; Strokoff et al., 2015). Additional research is warranted to examine personal meaning of the phrase “hooking up” in adolescent and non-college samples and how various definitions relate to short- and long-term positive and negative consequences.

The ambiguity of the term could be used in one’s favor so that one can create the desired perception of going further sexually than they had during the hookup or create the desired perception that one did not go as far as sexually as they had during the hookup (Bogle, 2007). Additionally, while there might be some consensus about the definition of hooking up, there is also the possibility that the definition varies or has an alternative form when it applies to the self and one’s relationships as opposed to others’ relationships (Epstein et al., 2009). Thus, adolescents and young adults may utilize the vagueness of the term as a way to either promote their sexual status or as a way to downplay their sexual experiences. While there are possible positive consequences resulting from the ambiguity of the term, there is also the potential for negative consequences. Ambiguity may increase the risk of negative emotional outcomes (Strokoff et al., 2015). Hooking up among adolescents or young adults often occurs in contexts where other people may have knowledge about one’s hookup or where participants disclose to others about the hookup (Spell, 2016). This could lead to others misinterpreting what was meant by the term hooking up, resulting in feelings of embarrassment, regret, and/or shame based on social reactions to the hookup.

Taken together, it is clear that although the term *hookup* lacks a single, universal definition, there appears to be some consensus among young people and researchers that hooking up involves sexual interactions that occur outside of committed romantic relationships (i.e., Heldman and Wade, 2010; Stinson, 2010; Garcia et al., 2012; Lewis et al., 2012; Claxton and van Dulmen, 2013). Similarly, although hookups may involve a wide range of sexual behaviors (e.g., kissing to penetrative sex) between partners who are not dating or in a romantic relationship, there is also some consenus that these interactions do not necessarily imply the start of a romantic commitment (Paul and Hayes, 2002; Epstein et al., 2009; Holman and Sillars, 2012; Lewis et al., 2012). Further, while providing a clear definition would help the field moving forward, a precise definition may obscure the intricacies of how individuals view their sexual behavior and relationship statuses. For example, when we label sexual behavior as “risky” or “beneficial,” what should be clear is what about the behavior is associated with risk (e.g., increased sexual transmitted infections; emotional suffering; esteem issues) and what can be “beneficial” (e.g., learning to be assertive in sexual situations; learning to respect partners’ expectations and desires). Thus, the ambiguity of this term has potential consequences related to health and well-being and has some potential limitations related to the interpretation and application of study findings.

## Alcohol and Hooking Up

As with sexual behavior more generally (Cooper, 2002, 2006; Leigh, 2002; Brown and Vanable, 2007; Cavazos-Rehg et al., 2007), research has shown hooking up to be associated with alcohol use at both global and event levels. The strength of the association between alcohol with hooking up varies by whether alcohol and hooking up are measured globally (i.e., alcohol consumption in an extended time frame, but not necessarily at the time of a hookup), at the situational level (e.g., alcohol use in the context of hooking up), or at the event level (i.e., alcohol consumption in a specific hookup encounter). Additionally, Claxton et al. (2015), in a meta-analysis of 29 studies, found a medium effect of alcohol use on hooking up, such that a higher levels of alcohol use were generally related to more hooking up (or casual sexual relationship experiences). For these reasons, clearly identifying the relationship between alcohol use and hooking up has been and continues to be a challenge.

### Global Associations Between Drinking Habits and Hooking Up

Alcohol consumption has been consistently linked to hooking up such that those who consume alcohol at high levels and use it more often are more likely than those who do not to engage in hookups overall, to hook up more frequently, and to report having a greater number of hookup partners (Desiderato and Crawford, 1995; Owen et al., 2010, 2011; Barriger and Vélez-Blasini, 2013; Olmstead et al., 2013a; Bernston et al., 2014; LaBrie et al., 2014; Roberson et al., 2015; Thomson et al., 2015). Although most research in this area has been conducted on college students, the general relationship between overall alcohol use and hooking up has been upheld in middle and high school students (Fortunato et al., 2010; Johnson, 2013). Research also indicates possible gender differences, such that the relationship between hooking up and alcohol use may be stronger for women than for men (Owen and Fincham, 2011; Owen et al., 2011). When examining specific hookup behaviors, findings vary according to the type of behavior assessed and the specific drinking measure. Some studies have found a relationship between binge drinking frequency and frequency of hookups involving either performing or receiving oral sex in first-year college women’s hookups (Fielder et al., 2013), between past month peak BAC and oral sex (Fielder and Carey, 2010a), and between typical drinking and oral sex (Lewis et al., 2012). However, one study found that general drinking habits did not predict giving or receiving oral sex (Barriger and Vélez-Blasini, 2013).

Findings are also mixed with regard to vaginal sex such that no relationship was found between vaginal sex and binge drinking in first-year students (Fielder et al., 2013), drinking frequency in students who had previously had at least one hookup (Gute and Eshbaugh, 2008), or overall drinking habits (Barriger and Vélez-Blasini, 2013). In contrast, Paul et al. (2000) found that undergraduates who reported the most alcohol-related symptoms when drinking, such as blurred vision, vomiting, and slurred speech – all of which indicate high consumption levels, were also most likely to have had a hookup that included sexual intercourse. Similarly, vaginal sex during hookups has been associated with typical drinking (Lewis et al., 2012) and past month peak BAC (Fielder and Carey, 2010a). A recent study found that among heavy drinking college students, drinking frequency was a largely consistent predictor of penetrative hooking up sexual behaviors (oral and vaginal sex) (Blayney et al., 2018). Together, this research indicates that the relationship may differ depending on what sort of sexual behaviors were engaged in during a hookup.

### Longitudinal Associations Between Alcohol and Hooking Up

In addition to these global studies, there have been few studies that examine longitudinal associations between alcohol and hooking up. Johnson and Chen (2015), using three waves (i.e., 1, 3, and 4) of the Add Health data, found that alcohol use (measured by a latent construct of alcohol use, binge drinking, and drunkenness) during the transition from ages 13–20 to ages 20–27 was associated with a greater number of hookups. Alcohol use during the transition period of 20–27 to 26–33 was a significantly stronger predictor of hookups than actual alcohol consumption during the ages of 26–33. Similarly, Johnson (2013), using the same three waves of the Add Health data as Johnson and Chen (2015), found that alcohol use in adolescence was a significantly stronger predictor of number of hookups in young adulthood than the pattern of alcohol use over time.

Additionally, there has been some longitudinal work examining college versus non-college-attending individuals. Vasilenko et al. (2017) found that for adolescents who did not eventually attend college, rates of non-relationship sex (i.e., sex with someone the participant was not in a relationship with) were significantly higher than for those adolescents who eventually attended college, including during the college years. The relationship between heavy episodic drinking and non-relationship sex was highest during the ages of 14–16 for both those who did and did not attend college later; however, the relationship at this age was stronger for those who eventually attended college. There were no significant differences between the two groups on the relationships between non-relationship sex and heavy episodic drinking between the ages of 15 and 18; however, at approximately age 18, the relationship between heavy episodic drinking and non-relationship sex increased for college attenders and remained higher than non-college attenders until around age 20 when significant differences between the two groups became non-significant. This indicates that there may be something specific about college attendance, and in particular, the first few years, that leads to increased rates of hooking up and alcohol use. One possible explanation is that since drinking occurs at relatively high levels during the college years (Wechsler and Nelson, 2008), the opportunity for sexual experiences while intoxicated is also higher. Thus, college students may represent a particular at-risk group whereby sexual decision making is often in the presence of alcohol.

In addition to the aforementioned, in one of the most comprehensive longitudinal studies on the relationships between alcohol and hookups, O’Hara and Cooper (2015) used both prospective (i.e., including only participants who had not had sex or who abstained from alcohol use) and trajectory analyses (using the complete sample) to examine the relationships between alcohol and both sex with a stranger and sex with a one-night stand. Their sample was drawn from a 15-year cohort study that began in adolescence (ages 13–19 at baseline) and followed participants until approximately age 30. Prospective analyses revealed that among those who were virgins at time 1, typical alcohol use (i.e., usual amount of alcohol consumed and frequency of drinking to intoxication) and heavy alcohol use (i.e., frequency of drinking until they become intoxicated and frequency of heavy episodic drinking) at time 1 predicted both sexual outcomes at time 2; however, alcohol use at time 1 was not related to sexual behavior at time 2. These relationships were not moderated by gender or race.

When utilizing the whole sample, trajectory analyses revealed that time 1 levels of alcohol use were related to initial levels of sex with a stranger and one-night stands. However, the divergence between those who were initially lower on alcohol use moved closer to their riskier counterparts over time, although the effects did not completely converge. Additionally, those who reported higher levels of alcohol use initially continued to have significantly more ocurrences of sex with a stranger into young adulthood. When examining these patterns by gender, men’s early alcohol use predicted sex with a stranger over time, but the effects diminished by young adulthood such that those who initially had lower levels of alcohol use became more similar in their sexual behavior to those who initially had high levels of alcohol use. For women, alcohol use during adolescence was largely unrelated to sexual behavior outcomes except for typical alcohol use and sex with a stranger. Women with higher initial levels of typical alcohol use were more likely to have sex with a stranger by their mid-20s. When examining these trajectories by race, Whites who had higher levels of typical alcohol use and higher levels of heavier drinking in adolescence engaged in sex with a stranger, both initially and linearly over time. For Blacks, the sizeable differences between initial levels of heavy drinking and the likelihood of having sex with a stranger were reduced by mid-20s. Additionally, Whites who had more alcohol problems engaged in one-night stands in a similar pattern as for sex with a stranger whereas this effect was not observed for Blacks. Further, baseline levels of sex with a stranger predicted increases in typical and/or heavy alcohol use as well as alcohol problems. Baseline level of having sex with a stranger was associated with higher initial level of typical and heavy alcohol use as well as with drinking problems; however, the growth of typical and heavy alcohol use slowed over time whereas the growth of drinking problems continued.

When examining these relationships by gender, only one interaction was present. The effect of sex with a stranger by time 1 was related to higher rates of alcohol problems for both men and women; however, for women, this effect disappeared over time while the effect persisted for men. For Blacks, a higher initial level of engaging in sex with a stranger was related to lower alcohol use until their early 20s. For Whites, sex with a stranger initially was related to both higher levels of typical and heavy alcohol use with effects diminishing over time by the early 20s.

### Event-Level Drinking and Hooking Up

One way to better understand the nature of the alcohol-hooking up association is to examine how they are related at the event level. Studies measuring alcohol consumption during specific hooking up events (i.e., event-level studies) are important for establishing temporal links between alcohol and hooking up behavior, representing a methodological improvement over global level studies. Between two-thirds and three-quarters of college students indicate drinking before hooking up (Grello et al., 2006; Downing-Matibag and Geisinger, 2009; Fielder and Carey, 2010b; Lewis et al., 2012; LaBrie et al., 2014). Both male and female college students indicate a greater likelihood of hooking up as opposed to traditional romantic dating if they have been drinking and drink more during hookups (Bradshaw et al., 2010; Fielder and Carey, 2010b). A commonly reported reason students provide for hooking up is drinking (Downing-Matibag and Geisinger, 2009; Kenney et al., 2013; LaBrie et al., 2014). Women on average have reported consuming an average of three drinks before a hookup (Fielder and Carey, 2010b) or as many as five (LaBrie et al., 2014) whereas in the only study assessing men’s drinking level, the average was seven (LaBrie et al., 2014). Other event level research indicates that college students, especially those who are single or who report hooking up, are more likely to engage in sexual behaviors on days when they used more alcohol relative to those who were in casual or committed relationships (Patrick et al., 2015; Wells et al., 2016). However, as with global association studies, relationships between event-level alcohol and sexual behaviors are not entirely consistent. For example, Lewis et al. (2012) found no relationship between number of drinks prior to a hookup and either oral or vaginal sex for men or women. Goldstein et al. (2007) found that oral sex was not associated with differences between a new sexual partner and a regular partner; however, drinking alcohol increased the likelihood of vaginal sex with a new partner compared to a regular partner. On the other hand, LaBrie et al. (2014) found a positive relationship between amount of drinking before a hookup and the degree of physicality engaged in if individuals had just met their partners on the night of the hookup. As we have presented, alcohol use and hooking up are linked; however, the direction of relations and (in)consistency of findings indicate that understanding the *process* whereby these two behaviors are linked requires substantial further investigation.

### Gender, Alcohol Use, and Hooking Up

While there is a relatively large literature examining gender (e.g., norms, double standards, affect) and hooking up broadly (e.g., Armstrong et al., 2012; Allison and Risman, 2013; Woerner and Abbey, 2017), there is less work examining these relationships in tandem with alcohol use. The general literature on women and sexual behavior indicates that for women it is difficult to navigate given the contradictory messages of self-development and empowerment, stigma surrounding hooking up behavior for women, gendered misperceptions that women always want a relationship, and the idea that refusing demonstrates that the woman has “standards” (i.e., not engaging in sex with men whom they did not want a romantic relationship with; Hamilton and Armstrong, 2009; Jozkowski et al., 2017). Further, too much casual sex is deemed negative, but *not* engaging in casual sex is also negatively perceived as a woman may be deemed prudish/uptight or a tease (Farvid et al., 2017; Jozkowski et al., 2017). This paradoxical position for women may lead to the need to “excuse” their behavior and one way may be through alcohol use as evidenced from qualitative studies (Lindgren et al., 2007; Farvid et al., 2017; Jozkowski et al., 2017). However, Lindgren et al. (2009) also found that women in their qualitative study highlighted that alcohol can also increase sexual assertiveness in both behavior and communication. Further evidence that alcohol and hooking up are linked among women come from studies that have found that, among women, motives and consequences for casual sex include alcohol use (Claxton and van Dulmen, 2013; Lovejoy, 2015).

Additionally, women are more concerned with safety and avoiding risk when searching for male hookup partners (e.g., fear of sexual assault; aggressiveness; Kuperberg and Padgett, 2015). Women are also concerned when a man is drinking and their concern is not only for themselves as they are also concerned with safety when the group of women who they are out with is also all drinking (Lindgren et al., 2009). Further, it is not only that women perceive greater risk when alcohol is involved, men echo the idea that they are more aggressive when drinking than they normally would be without alcohol (Lindgren et al., 2009); however, the perception that men become more aggressive under the influence of alcohol is normative in hegemonic masculinity.

Men also acknowledge the double standards surrounding hooking up and gender (e.g., status; ambivalence of women’s sexual behavior; dominance; stigma; Sweeny, 2014) as well as the disrespectful and demeaning treating of women and sexual objectification (Kalish, 2013; Sweeny, 2014). These double standards are implicated in rape myths (e.g., male aggressiveness in sexual relations; men’s sexual drives are driven by biology and are uncontrollable; ambiguity in situation; perpetrator justification; trivialization of sexual assault) and victim blaming (Becker and Tinkler, 2015; Farvid et al., 2017) as well as sexual victimization (Lovejoy, 2015) especially when alcohol is involved (Becker and Tinkler, 2015), which seem to be related to hooking up for both men and women (Reling et al., 2018). Overall, disentangling gender stereotypes and norms, alcohol use, and hooking up still needs substantial development.

## Why Are Alcohol and Hooking Up Linked?

The preponderance of evidence suggests that consuming alcohol, especially at high levels, is related both to the occurrence of hooking up and to a variety of outcomes, such as negative emotionality, increased risk of unwanted sex, including sexual assault, and exposure to sexually transmitted infections. From an alcohol myopia standpoint (Steele and Josephs, 1990), alcohol use and hooking up are related as alcohol disinhibits via effects on information processing. Within the broader context of alcohol myopia theory, research indicates that several different factors, described below, may be more or less salient and likely to be attended to during times of sexual decision when alcohol is involved.

### Sexual Scripts

Sexual scripts are cognitive schema that instruct people how to understand and act in sexual situations (Simon and Gagnon, 1984; Masters et al., 2014). According to research by Paul and Hayes (2002), the typical script for hookups involves parties where interactions with potential partners are facilitated by alcohol. Research indicates that alcohol use is frequently mentioned as an important part of sexual scripts related to hooking up among college students. In particular, research has highlighted the relevance of a near-campus party or bar scene because the consumption of alcohol and “partying” are central to the hookup script (Paul and Hayes, 2002; Bogle, 2008; Allison and Risman, 2014; Eaton et al., 2016) with some work indicating that over 60% of students mentioned alcohol as being a key part of hooking up sexual scripts (Holman and Sillars, 2012). Research examining hookup scripts described in young adults’ most recent hookup encounters found that 31.2% explicitly mentioned alcohol use while 16.2% implied the presence of alcohol with only 1% overtly stating that alcohol was not a factor in their most recent hook-up (Olmstead et al., 2018). While drinking, it may be that individuals default to these sexual scripts related to hookups, which may make them more likely to drink alcohol before or during a hookup, which in turn can result in alcohol myopia whereby individuals pay more attention to the impelling cues (i.e., sexual arousal) around them rather than thinking about long-term consequences (i.e., STIs) associated with behavior.

### Contextual Factors

Another possible explanation behind the relationship between alcohol use and hooking up may be that those who drink heavily in general are those who drink heavily in situations where hooking up is likely to occur indicating that contextual factors are likely important in promoting hooking up behaviors. College students commonly meet hookup partners at bars, parties, or other events that encourage alcohol consumption, often at excessive levels (Paul and Hayes, 2002; Bersamin et al., 2012) and may even seek out drinking contexts in order to find a sexual partner (Lindgren et al., 2009). For example, Kuperberg and Padgett (2017) found that for college students, meeting in bars or at parties through common interest groups or history and (for women) at dormitories was associated with binge drinking during hookups; meeting online and (for women) in public was associated with reduced heavy drinking during encounters. Related, research by Dai et al. (2017) found that college students’ beliefs that attending parties and events enable hooking up behavior were positively associated with hooking up behavior. Other work with adolescents found that adolescents who met their partner in a public place (compared to school) were more likely to have used alcohol prior to sex (Staras et al., 2012). For women, alcohol-involved first coitus was more likely to occur with a casual partner and in the context of a party or social gathering (Livingston et al., 2015). One possible explanation for the importance of context is that certain situations present triggers or cues related to social norms and expectations about engaging in some level of sexual activity (Fielder and Carey, 2010a). Further, when individuals have been drinking, these contextual triggers or cues may be particularly salient and may play a larger role in decisions to engage in hooking up.

### Expectancies

Alcohol-related expectancies may play an especially significant role in motivations to hook up among adolescents and young adults. Research has indicated that college students strongly associate alcohol consumption with participating in some type of sexual activity (Lindgren et al., 2009). Furthermore, students who have high alcohol expectancies about sexual risk-taking or disinhibition may be especially vulnerable to engage in behaviors that would put them at risk for contracting sexually transmitted infections after drinking (LaBrie et al., 2005). Recent research has found evidence supporting both a mediating and a moderating role of expectancies. Sexual but not social enhancement drinking expectancies moderated the association between hookup intentions and heavy drinking (Beckmeyer, 2017) whereas social/physical pleasure alcohol expectancies mediated the relationship between hooking up and drinking behavior (Tyler et al., 2017a, b). When drinking, these alcohol expectancies may be at the forefront of individuals’ thinking (i.e., serve as impelling cues such that individuals focus more on the expectation of sexual pleasure than potential risk) and can thus guide their decision-making regarding hooking up.

### Motivations

In addition to expectancies, research indicates that people may use sexual behavior to meet certain psychological needs (i.e., Cooper et al., 1998; Grossbard et al., 2008; Patrick et al., 2011), and researchers have examined how alcohol and hooking up may be related to various sex-related motivations. Dvorak et al. (2016) found that both social motives (i.e., drinking to be more social) and enhancement motives (i.e., drinking to feel the pleasurable effects of alcohol) were indirectly associated with the likelihood of hooking up via alcohol use. Those who reported drinking to experience the pleasurable effects of alcohol or drinking to become more social exhibited higher levels of alcohol use, which in turn increased the likelihood of hooking up. More recent work found that enhancement motives predicted more frequent oral and vaginal sex when hooking up whereas peer and partner motives (i.e., having sex to please one’s partner or to gain approval) predicted anal sex while hooking up (Blayney et al., 2018). This same study also noted gender differences; relative to women, men reported higher enhancement and peer motives when hooking up, and for men in particular coping motives (i.e., having sex to deal with negative emotions) were associated with more frequent oral and vaginal sex, and peer motives were associated with more frequent anal sex when hooking up (Blayney et al., 2018). Taken together, this research indicates that certain motivations may be implicated when examining hooking up among heavy drinkers and that a better understanding of how alcohol-related hooking up meets particular psychological needs may inform and improve prevention strategies.

### Technology, Hooking Up, and Alcohol

An emerging trend in the literature surrounding alcohol use and hooking up is related to the role that technology plays. Technology, like text messaging and dating apps, has provided new channels through which hooking up and alcohol use may be facilitated. Sexting is a unique blend of technology and sexual interaction that is growing in popularity and may be an important mechanism in explaining risk for sexual hookups (Dir et al., 2013). Sexting is typically defined as the act of sending sexually explicit or suggestive photographs via text message (Diliberto and Mattey, 2009; Jolicouer and Zedlewski, 2010). A growing literature has indicated that, among college students, sexting and alcohol use are related (e.g., Benotsch et al., 2012) with some tentative findings among adolescents (Dake et al., 2012; Temple et al., 2014). Other studies have found that sexting predicts sexual hookups over and above alcohol use (Benotsch et al., 2012; Dir et al., 2013), suggesting that sexting could be a partial mediator in the relationship between alcohol use and sexual hookups. In addition to sexting, some research has begun to examine how the use of online “hookup apps” (e.g., Tinder) may be related to alcohol use and actual hooking up behaviors. Research has confirmed that hooking up is a key motivation for app use among Tinder users (Carpenter and McEwan, 2016; Gatter and Hodkinson, 2016), and it has been suggested that some college students report drinking prior to use of such hooking up apps (Sager et al., 2016). Drinking prior to using a hooking up app may be another way in which alcohol myopia comes into play, whereby the inhibiting and impelling cues an individual is exposed to are entirely online. For example, someone who is drinking and searching on a hooking up app may pay more attention to cues like attractiveness of the person in the profile versus considering for a hook up the risk of meeting someone that they do not know. Although there has been significant progress related to technology, alcohol, and hooking up, this work is still preliminary in nature and additional work examining alcohol use, hooking up, and apps is warranted and much needed in order to better understand this emerging phenomenon.

## Outcomes Related to Alcohol-Involved Hookups

In addition to the investigations of predictors of alcohol consumption and hookups, several research studies have explored the positive and negative consequences of alcohol-involved hookups. Although students often express positive feelings about their hookups (Paul and Hayes, 2002; LaBrie et al., 2014; Snapp et al., 2015), alcohol-involved hooking up may put them at risk for a number of negative outcomes. Regret or feelings of discontent are common after alcohol-involved hookups, perhaps because many students, especially women, report that they would not have gone as far during the hookup if they had not been drinking (Downing-Matibag and Geisinger, 2009; LaBrie et al., 2014). Lewis et al. (2012) found that increased drinking at the time of a hookup was related to lowered positive affect and heightened negative affect afterward for both men and women. Alternatively, a study of male and female undergraduates used cluster analysis and found four types of hookup reactions. The group with the most positive reactions (the “Happy Hopefuls”) reported greater intoxication at the time of the hookup than did the other three clusters (Strokoff et al., 2015).

Some studies have found that alcohol-involved hookups may evoke different emotional reactions for men and women. For example, LaBrie et al. (2014) found that women who had consumed alcohol at the time of the hookup reported greater discontentment afterward than women who had not consumed alcohol, but did not find significant differences for alcohol consumption in discontentment among men. Other studies, however, have not found such gender differences. Lewis et al. (2012) found that predictors of greater positive affect after hookups (e.g., more positive hookup attitudes and greater approval of hookups) and predictors of greater negative affect after hookups (e.g., number of drinks during the hookup, vaginal sex during the hookup, and relationship with the hookup partner) did not differ by gender. Owen and Fincham (2011) similarly reported that gender did not interact with any of the predictors of positive and negative emotional reactions. In their study, alcohol use during the hookup was predictive of less positive and more negative emotional responses for both men and women. In one study of female undergraduates, alcohol consumption during the hookup was unrelated to negative reactions, sexual/romantic reactions, and social/academic engagement reactions when considered in conjunction with other predictors (Owen et al., 2013). Overall, findings are quite mixed regarding the positive and negative emotional consequences of alcohol-involved hookups and whether these consequences differ for men and women, which is a clear limitation of the current literature.

It should be noted that consequences of alcohol use and hooking up are not limited to emotional ones. For example, research indicates that there may be a social cost associated with drinking and hooking up. Experimental research using a series of vignettes indicated that when the person in the vignette hooked up while drunk, they were perceived less favorably (i.e., being of poor character, less likeable, less responsible) than peers who did not engage in both of these behaviors (Penhollow et al., 2017). In addition to social consequences, health consequences may also occur after alcohol-involved hookups. Individuals may be at increased risk of contracting STIs if they hookup after drinking heavily. Although Lewis et al. (2012) did not find a relationship between consuming alcohol at the time of a hookup and condom use, in other studies students attributed their non-use of a condom during hookups to having been intoxicated (Desiderato and Crawford, 1995; Downing-Matibag and Geisinger, 2009). Given the health risks (e.g., sexually transmitted infections, unplanned pregnancy) conferred by unprotected sex, additional research regarding alcohol’s impact on condom use likelihood during hookup situations is clearly warranted. Alcohol myopia has been implicated as a reason for condom non-use during sexual encounters more broadly (see Rehm et al., 2011, for a review); thus, it is not surprising that it may also be implicated in hooking up more specifically.

Beyond the aforementioned consequences, a particularly distressing experience related to drinking at the time of hooking up is unwanted sexual activity (Stinson, 2010). Although some individuals might consent to low-level acts, such as kissing, excessive intoxication might lead to the inability to remove oneself from an uncomfortable situation (Paul and Hayes, 2002) or to resist unwanted fondling and intercourse effectively (Paul and Hayes, 2002; Flack et al., 2007; Downing-Matibag and Geisinger, 2009). Hookup behaviors and heavy alcohol consumption are associated with increased risk of sexual victimization in college women (Testa et al., 2010), and 18% of women who had an alcohol-involved first coitus experience reported that it was without consent or against their will (Livingston et al., 2015). Moreover, sexual assault often occurs within the hookup context. For example, a study by Flack et al. (2016) demonstrated that the majority (77.6%) of sexual assaults reported by a female college student sample occurred during a hookup. Although this study found that general alcohol consumption was positively related to both hooking up engagement and sexual assault victimization, it was unclear the extent to which alcohol had been consumed in the situations involving sexual assault during a hookup. Examining several factors related to sexual assault during the most recent hookup, including alcohol use, Ford (2017) found that among heterosexual women at 4-year colleges/universities (*n* = 8,005), women who drank upward of nine drinks were more likely to experience physically forced intercourse during their most recent hookup than women who drank less. Additionally, women who drank three or more drinks were more likely to experience incapacitated sexual assault compared to women who had not drank during their last hookup (Ford, 2017). Similar cross-sectional survey findings were reported by Tyler et al. (2017a) with both men’s and women’s sexual victimization being positively associated with hooking up, heavy drinking, and alcohol expectancies. However, as with other studies, event-level associations among these variables were not examined which is a limitation.

## Intervention

Given that adolescents and young adults indicate serious short- and long-term negative consequences of hooking up, there is a need for future research to develop and evaluate hooking up intervention content to reduce negative consequences from hooking up. Current interventions focusing on alcohol use or sexual risk taking do not include content specific to hooking up or have yet to evaluate intervention content on hooking up behavioral outcomes (Dermen and Thomas, 2011; Lewis et al., 2014). Although there is a great deal of evidence that brief normative feedback interventions can reduce alcohol use (Tanner-Smith and Lipsey, 2015) and some evidence such interventions may prevent alcohol-related risky sexual behavior (Lewis et al., 2014, 2018), research related to brief interventions for hooking up is limited. We are aware of only one study (Testa et al., submitted) that has tested and found evidence for the efficacy of brief normative feedback interventions for hookups. This work (Testa et al., submitted) provides evidence for the importance of hookups as a risk factor for sexual victimization and provides preliminary support for an intervention that alters descriptive norms as a way of reducing hookups and consequently sexual vulnerability.

As mentioned previously, future intervention research should consider how alcohol use, hooking up, and sexual victimization are associated. Interventions should consider that vulnerability for sexual victimization can result from female intoxication or incapacitation within a setting in which a potential perpetrator is present, such as within sexual situations or “hookups” (Paul and Hayes, 2002). Having more sex partners or hookups increases the odds of sexual victimization (Messman-Moore et al., 2008; Franklin, 2010; Tomsich et al., 2013; Fielder et al., 2014), and hooking up is a risky context for sexual victimization (Paul and Hayes, 2002; Flack et al., 2007; Lewis et al., 2012). Other recent work indicates that more frequent heavy drinking is associated with increased risk for subsequent incapacitated rape, but only among bisexual women who reported more than one male hookup partner. Therefore, it may be particularly important to target hooking up interventions to reduce sexual victimization among bisexual women (Jaffe et al., submitted). In sum, because hooking up provides a risky context for sexual victimization, in particular alcohol-involved sexual victimization, intervention content should consider the interrelations among alcohol use, hooking up, and sexual victimization. Reducing hooking up occurrences may also reduce related risks, such as sexual victimization, as perpetrators can use hookups to gain access to potential victims.

To date, experimental research has begun to examine the protective role college students’ peers can play and the situational factors that might influence willingness to intervene when a close friend is about to hookup when intoxicated (Savage et al., 2017). Savage et al. (2017) found that participants intended to intervene with female friends, but not male friends, and women were more likely to intervene than men. Their findings also indicated that sober participants had stronger intentions to intervene than intoxicated ones, but this effect was driven by increases in men’s intentions when sober.

Specific to the alcohol myopia framework, several studies examining sexual behavior broadly (i.e., not specific to hooking up) have found that health intervention programs may be more efficacious if they include strategies such as reminder cues to increase the salience of health information in appropriate contexts (MacDonald et al., 2000; Dal Cin et al., 2006). These findings are consistent with alcohol myopia theory (Steele and Southwick, 1985; Steele and Josephs, 1990), such that when cues promoting safe sex were made salient, intoxicated individuals were more likely to attend to those cues. Despite this work, we are unaware of any study specifically looking at cue salience interventions for hooking up behaviors when intoxicated. Given event-level research linking the two, interventions that capitalize on in-the-moment decisions when drinking may lead to reductions in hooking up in these situations. As such, increasing the salience of reminder cues for safer sex when drinking and hooking up is an important area for future study and might lead to the development of relatively brief interventions.

## Future Directions in Research

Despite the growing knowledge in understanding the relationships between and consequences of alcohol and hooking up, it should be noted that there are still several important unexplored or marginally explored directions that warrant further development. First, as highlighted in reviews focused on hooking up behavior (e.g., Heldman and Wade, 2010; Pham, 2017; Watson et al., 2017), much of the research to date on hooking up has been focused on predominantly White, heterosexual, class-privileged college students. There is still a dearth of studies on other groups (e.g., adolescents, older adults, non-college attending young adults, ethnic, and sexual minority groups) and even fewer that investigate the role of alcohol use. For example, there is little known research on hooking up and alcohol use among adolescents or longitudinal studies that bridge adolescence and adulthood. Moroever, the studies that do exist are primarily not conducted in the United States (e.g., Dubé et al., 2017); thus, little is known about the generalizability of the findings to United States adolescents. Additionally, understanding the transitions across time from adolescence into young adulthood among those who intend or do attend college compared to those who do not intend or go to college is also an important avenue for development. As demonstrated by Vasilenko et al. (2017), individuals who do not attend college had higher rates of non-relationship sex than those who attended college; however, the relationships between heavy episodic drinking and non-relationship sex was higher among those who attended college prior to college attendance (i.e., ages 14–15) and in the early stages of college (i.e., ages 18–20). These differences disappeared by age 20, and there were no subsequent significant differences between the groups. Therefore, alcohol and hooking up may represent a bidirectional relationship that seems to intensify in certain contexts among certain individuals. Although alcohol myopia theory provides a theoretical basis for understanding how alcohol use can lead to hooking up, the bidirectional nature of hooking up leading to increased alcohol use indicates that more complex associations outside of the theory of alcohol myopia should be investigated.

There are also other venues for generalizability to be improved. For example, only a few notable studies that examine the alcohol and hooking up relationship have included examinations of race or ethnicity (see Claxton et al., 2015, for a review). There is also little information on hooking up behavior among sexual minorities. Despite indications that lesbian, gay, and bisexual individuals hook up at high rates (Watson et al., 2017), with some studies finding that lesbian, gay, and bisexual individuals hook up more than their heterosexual counterparts (Hall et al., 2017), there is even less information on the hooking up and alcohol relationship for these groups. Outside of a few notable studies (Allison and Risman, 2014; Spell, 2016), there is an absence of research on intersectionality of identities and the hooking up – alcohol relationship, regardless of its shown importance. Related, there is a need for more nuanced examinations of the alcohol and hooking up relationship by gender. As mentioned previously, despite the literature examining different aspects related to gender (e.g., norms, double standards, affect) and hooking up broadly (e.g., Woerner and Abbey, 2017), research is less established related to how gender may impact the relation between alcohol use and hooking up. Further, public drinking spaces and alcohol consumption, coupled with gender and alcohol norms, have been implicated in both sexual victimization (Kavanaugh, 2013) and sexually aggressive experiences (Becker and Tinkler, 2015). It should be noted that there is little work that focuses on hooking up and unwanted sexual contact with men as the victims and how masculinity norms affect men’s hookup behavior or ambivalence of continuing sex acts within hookup occurrences (see Kalish, 2013; Danube et al., 2014; Ford, 2017; for exceptions), especially in conjunction with alcohol use. Further, despite the limited research on consent, alcohol, and hooking up, researchers have not yet followed up with more in-depth studies of how hooking up and alcohol use may lead to sexual assault. Even though unwanted sexual activity during hookup events is not typically labeled as rape or sexual assault by the victim (Flack et al., 2007; Downing-Matibag and Geisinger, 2009), future research should examine if they lead to the same types of post-sexual assault distress as experiences that are labeled as rape by the victim and how alcohol’s involvement might influence such reactions. Moreover, because the responsibility for sexual assault during an alcohol-involved hookup lies with the perpetrator, future investigations should explore the ways in which intoxicated individuals are targeted by perpetrators in hookup contexts.

One of more important gaps in the literature is that little is known about concurrent or simultaneous other drug use and alcohol use within hooking up contexts. The studies that measure alcohol and other drug use tend to not disentangle the effects of multiple substances or do not evaluate the possible synergistic effects. The most developed studies examining alcohol and other drug use frequently focus on marijuana use with few examining synergistic effects of alcohol and marijuana use (e.g., Metrik et al., 2016). Some studies ask vague questions such as whether any alcohol or drugs were used at the time of the hookup (Paul and Hayes, 2002; Grello et al., 2006), make composite scores of drug and alcohol use (Bailey et al., 2011), or focus on a composite “risky sex” variable, which may or may not be applicable to hooking up (Simons et al., 2010). Notably, research examining specific substances other than marijuana and alcohol during hookups is minimal, which is surprising given that some substances in particular have been associated with increased likelihood of sexual activity (Theodore et al., 2014; McKetin et al., 2018). Much more specific knowledge is needed about for whom, how, and which drugs may be used in conjunction with alcohol and hooking up.

Finally, with the rise of technology and hooking up/meeting apps, further investigations focused on the intersection of hooking up, alcohol use, and technology is warranted. Because there are dating and meeting apps not only for young adults, but for adolescents as well (e.g., myLOL, skout, blendr), additional research is needed in this area. While previously men who have sex with men represented the largest proportion of geospatial networking apps (GSN; e.g., Tindr), 7% of cell phone app users have used a GSN dating app in their lifetime (Smith and Duggan, 2013). Further, use rates between young adult men and women are becoming more comparable (e.g., Hahn et al., 2018). However, little is known about alcohol and hooking up related to app use, outside of general drug and alcohol risk among men who have sex with men or in non-United States samples (e.g., Choi et al., 2017). Additionally, with the advent of sexting and its relationship to alcohol use and hookups (Dir et al., 2013), as well as findings between online sexual disclosures relating to hooking up offline and alcohol use (Bobkowski et al., 2012), more research on the use of technology for hooking up behaviors and how alcohol is related is warranted.

## Conclusion

A wealth of research has accumulated over the past 15 years as the term hooking up has emerged in the literature. However, this review has noted that there are still a number of important unanswered questions related to hooking up and alcohol use. Despite the relative dearth of research on long-term emotional and health outcomes of alcohol-related hookups, as well as methodological limitations of the existing research (e.g., limited samples and mostly cross-sectional research designs), health promotion efforts are still warranted. Such programs should aim to minimize the harms that alcohol-related hookups might cause, help adolescents and young adults identify and communicate their expectations related to hooking up and monitor barriers to making good relationship decisions (e.g., alcohol and other substances). This work is particularly important given that we know alcohol consumption can lead to poorer judgment and decision making (Steele and Josephs, 1990). As such, health promotion efforts that utilize technology (e.g., cell phones; apps) might be one way to reach individuals in situations when they are at higher risk for using alcohol and hooking up.

Although the field has made significant progress, there is still much work to be done in order to increase our understanding of how hooking up and alcohol use relates to emotional consequences, sexual behavior, and personal and cultural characteristics of adolescent and young adults. A more thorough and nuanced understanding of when and for whom the relationship between alcohol and hooking is strongest and how these encounters may differentially predict health-related outcomes has the potential to inform the development of preventative interventions to reduce the risks associated with alcohol use and hooking up.

## Author Contributions

TG was in charge of writing several sections, reviewing the manuscript sections, collecting and organizing the relevant literature for the co-authors, and overall editing of the manuscript. DL and ML assisted the first author in writing several sections, reviewing the manuscript sections, and overall editing of the manuscript. KD, JN, and DK assisted the first author in writing several sections and overall editing of the manuscript.

## Conflict of Interest Statement

The authors declare that the research was conducted in the absence of any commercial or financial relationships that could be construed as a potential conflict of interest.
